# Surface Engineering of ZnO Thin Film for High Efficiency Planar Perovskite Solar Cells

**DOI:** 10.1038/srep13211

**Published:** 2015-09-28

**Authors:** Zong-Liang Tseng, Chien-Hung Chiang, Chun-Guey Wu

**Affiliations:** 1 Research Center for New Generation Photovoltaics, National Central University, Jhong-Li, 32001, Taiwan, ROC; 2 Department of Chemistry, National Central University, Jhong-Li, 32001, Taiwan, ROC

## Abstract

Sputtering made ZnO thin film was used as an electron-transport layer in a regular planar perovskite solar cell based on high quality CH_3_NH_3_PbI_3_ absorber prepared with a two-step spin-coating. An efficiency up to 15.9% under AM 1.5G irradiation is achieved for the cell based on ZnO film fabricated under Ar working gas. The atmosphere of the sputtering chamber can tune the surface electronic properties (band structure) of the resulting ZnO thin film and therefore the photovoltaic performance of the corresponding perovskite solar cell. Precise surface engineering of ZnO thin film was found to be one of the key steps to fabricate ZnO based regular planar perovskite solar cell with high power conversion efficiency. Sputtering method is proved to be one of the excellent techniques to prepare ZnO thin film with controllable properties.

Methylammonium lead halide (CH_3_NH_3_PbX_3_, X = I, Cl or Br) has attracted extensive attention because of its promising light-harvesting capability. Solid-state dye-sensitized solar cell based on perovskite sensitizer and mesoporous metal oxide scaffold (conventional perovskite solar cell) achieves the conversion efficiency over 17%^1^,^2^,^3^,^4^. Now solid state perovskite solar cells has reached the certified power conversion efficiency over 20%^5^,^6^. In a conventional DSSC, mesoporous semi-conducting metal oxides are used to adsorb a large number of dye molecules for absorbing the light and injecting electron to the anode to produce the electricity. However, insulated mesoporous Al_2_O_3_ scaffold layer without electron accepting ability was also developed in perovskite sensitized solar cells and demonstrated a PCE of up to 10.9%^7^. Moreover, it was shown that perovskite materials which have low exciton binding energy and long carrier lifetime thus can be also applied in the planar heterojunction solar cells^8^,^9^ in which the mesoscopic oxide scaffold layer is no longer required to achieve high efficiency. The merit of the planar (without meso-superstructure) architecture is that it can be adopted by the low temperature solution process and therefore the flexibility device^10^,^11^,^12^,^13^,^14^.

In a planar perovskite-based solar cell, a metal oxide dense film, such as TiO_2_^8^,^9^ or ZnO^15^,^16^,^17^ is usually used as an electron-transport layer to beneficially transfer the electrons and block the holes. The thin metal oxide layer can be regarded as a modification agent to lower the work function of the modified ITO anode^18^,^19^,^20^. It is well known that the intrinsic point defects, such as oxygen vacancy and metal interstitial, have important impacts on the electronic properties of the metal oxides. It has been also commonly accepted that the presence of oxygen vacancies (non-stoichiometry or zinc interstitials) in ZnO can increase the charge density of ZnO to form native n-type semiconductor^21^,^22^,^23^. However, up to date, few reports discussed the effects of the non-stoichiometry and defects of the metal oxide electron transport layer on the photovoltaic performance of the planar perovskite solar cell.

In this study, we report the photovoltaic performance of the planar CH_3_NH_3_PbI_3_ perovskite solar cell based on ZnO electron-transport layer prepared by sputtering deposition method. The stoichiometry and therefore the properties of ZnO thin film was controlled by adjusting the ratio of working gases (Ar and O_2_) in the vacuum chamber during radio frequency (RF) magnetron sputtering process. The effects of ZnO (thin film prepared under different working gas environments) properties on the photovoltaic performance of the corresponding perovskite based solar cell were investigated. The highest efficiency up to 15.9% (under AM1.5G irradiation) was achieved for the cell based on ZnO electron transport layer prepared under pure Ar working gas. The results provide the useful information regarding to the chemical engineering of the electronic (band structure) and electric (conductivity) properties of the electron transport layer to enhance the photovoltaic performance of the planner perovskite solar cell.

## Results and Discussion

**Figure 1:**
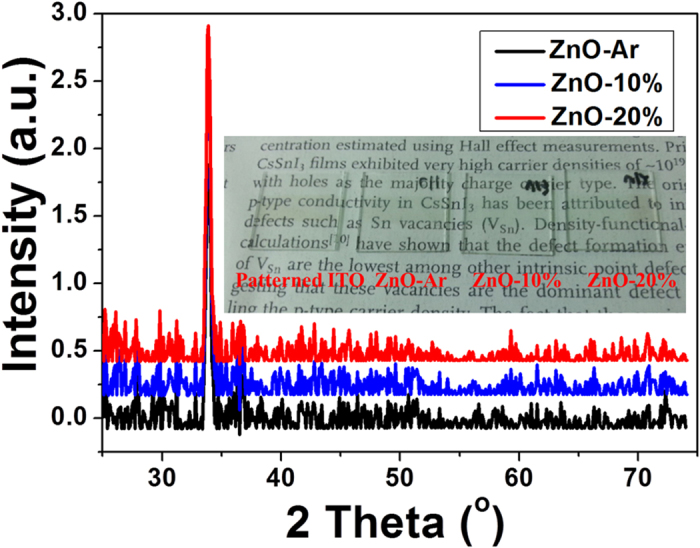
X-ray diffraction patterns of ZnO films deposited on ITO substrates prepared by sputtering under working gas of pure Ar (ZnO-Ar) and Ar/O_2_ mixture (ZnO-10% and ZnO-20%). (The inset shows a photograph (taken by Z.-L. Tseng) of a commercial patterned ITO substrate and ZnO films deposited on ITO substrates).

The deposition rates for preparing ZnO films under pure Ar (ZnO-Ar), O_2_/Ar + O_2_ = 10% (ZnO-10%), and O_2_/Ar + O_2_ = 20% (ZnO-20%) were 1.9, 1.3, and 1.3 nm/min, respectively. The thickness of all films was controlled to be *ca.* 40 nm by adjusting the deposition time. X-ray diffraction patterns of all ZnO films on the glass substrates prepared by sputtering under various working gases without substrate heating were displayed in Fig. 1. The pictures of the as-deposited (with or without the presence of O_2_) ZnO films are displayed in the insert of Fig. 1. All ZnO films are highly transparent and the optical band-gap estimated from the Uv/Vis absorption spectra (displayed in Figure S1 of the Electronic Supporting Information, ESI) is 3.2 eV for all films, consistent with the high band-gap semi-conductor characteristics. The diffraction patterns of ZnO-Ar, ZnO-10%, and ZnO-20% films are also similar. Only (0 0 2) diffraction peak appeared at 2θ of 34° was observed indicating that all ZnO films showed a preferential orientation with the c-axis perpendicular to the substrates. Furthermore the diffraction peak for all films has similar intensity and line width suggesting that the crystallinity and crystallite size of ZnO films grown under different working gases are very close with each other.

**Figure 2:**
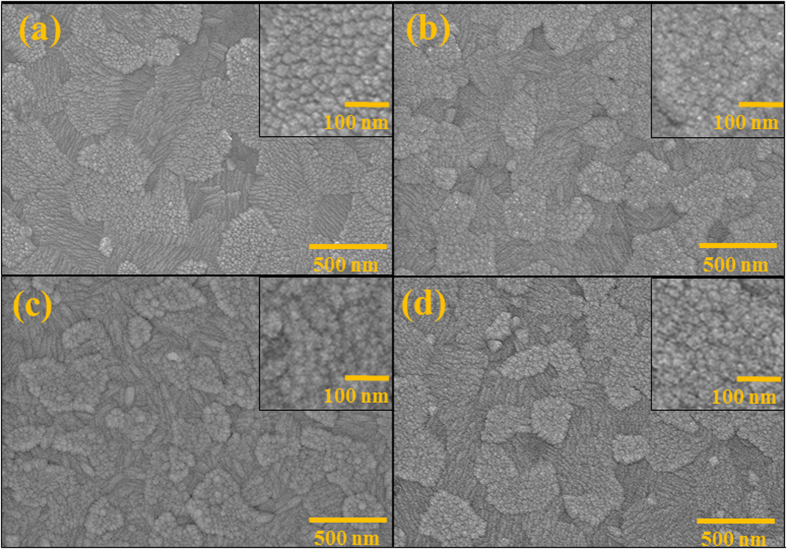
SEM images of (**a**) a commercial ITO substrate, (**b**) ZnO-Ar film, (**c**) ZnO-10% film, and (**d**) ZnO-20% film coated on the ITO substrate. The inset in each picture shows the same images at higher magnification.

SEM images of ITO and ZnO films deposited on ITO substrates were shown in Fig. 2. ITO film (Fig. 2(a)) was consisted of very homogeneous grains with the particle size of *ca.* 20 nm. ZnO films were formed by the aggregated nano-crystals having the particle size less than 10 nm, laying on the top of ITO grains, as shown in Fig. 2(b–d). The morphologies of ZnO films prepared under different working gases are also similar, may be due to both films are very thin (40 nm) and using the same batch of substrate (ITO).

**Figure 3:**
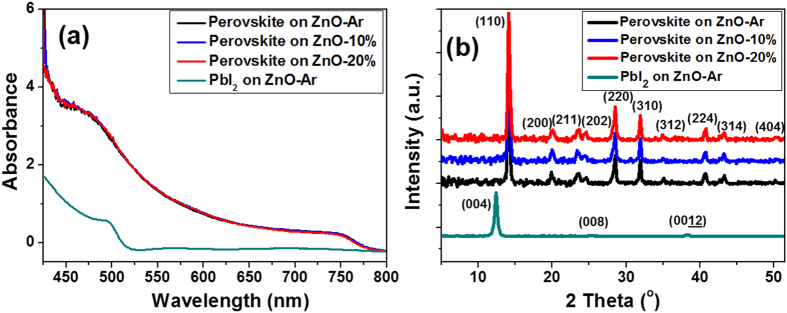
(**a**) Absorption spectra and (**b**) XRD patterns of PbI_2_ film and CH_3_NH_3_PbI_3_ films deposited on top of ZnO films.

CH_3_NH_3_PbI_3_ film deposited on the top of ZnO film was prepared with a two-step method we developed recently using solution process at low temperature^24^. The absorption spectra of CH_3_NH_3_PbI_3_ films displayed in Fig. 3(a) shows a strong absorption at the wavelength between 400 nm to 800 nm, which has the ability to harvest the visible light. The absorption coefficients (absorption divided by film thickness) of the CH_3_NH_3_PbI_3_ films deposited on ZnO-Ar, ZnO-10% and ZnO-20% were 3.8 × 10^4^, 3.9 × 10^4^ cm^−1^ and 3.9 × 10^4^ cm^−1^ at 550 nm, respectively. The result revealed that perovskite films deposited on all ZnO films have similar absorption intensity and they all are the highly dense film^25^,^26^ (The pictures of PbI_2_ on ZnO-Ar, and perovskite films deposited on the top of ZnO-Ar, ZnO-10% and ZnO-20% film were displayed in Figure S2 of ESI). Figure 3(b) is the GIXRD patterns of perovskite films deposited on three different ZnO films and the XRD pattern for PbI_2_ was also listed for the comparison. Eleven diffraction peaks corresponding to the (110), (200), (211), (202), (220), (213), (310), (312), (224), (314), and (404) planes of the CH_3_NH_3_PbI_3_ were observed^27^ and no peak belongs to PbI_2_ was detected. The crystallinity and crystalline domain size of CH_3_NH_3_PbI_3_ film deposited on ZnO films made from different working gases are also very alike, due to all perovskite films were prepared with nearly the same conditions.

**Figure 4:**
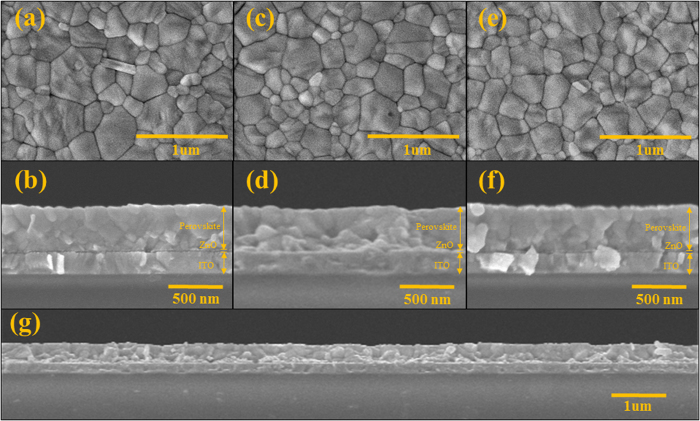
The surface and cross-sectional SEM images of perovskite films deposited on ZnO-Ar film (**a**,**b**), deposited on ZnO-10% film (**c**,**d**), deposited on ZnO-20% (**e**, **f**) and (**g**) is the cross section image at low magnification of perovskite deposited on ZnO-Ar to reveal the long-range homogeneous of perovskite film.

Figure 4 illustrates the surface and cross-section SEM images of perovskite film deposited on ZnO-Ar, ZnO-10% and ZnO-20% films. The surface morphology of CH_3_NH_3_PbI_3_ films are also very alike, exhibiting a dense-packed grains with the sizes of about 100 ~ 500 nm. Their morphology is similar to those fabricated with solvent-engineering technology^28^ and vapor-assisted solution process^29^ reported previously. Dense and continuous perovskite film is essential for achieving high power conversion efficiency of the resulting cell. Two-step spin-coating method is a way to fabricate dense perovskite film to be applied in the planner heterojunction device when the electron transport layer with relatively smooth surface was used^24^. The thickness and packing of the grain of the active perovskite film which acts as a light harvester is also an important parameter for determining the photovoltaic performance of the corresponding device. Figure 4(b,d,f) display the cross-section SEM images of perovskite films coated on top of ZnO-Ar, ZnO-10%, and ZnO-20% films. The thickness of perovskite films is all about 350 nm and very dense without observable pine hole. It was known^6^,^7^ that perovskite absorber has long charge carrier lifetime and exciton diffusion length. Therefore, perovskite film with the thickness of 350 nm was able to harvest light sufficiently and transport the carriers (or excitons) to the electrode efficiently. Figure 4(g) is a low magnification of the cross-section image which reveals that the film is very flat and dense in a long distance (>10 um).

**Figure 5:**
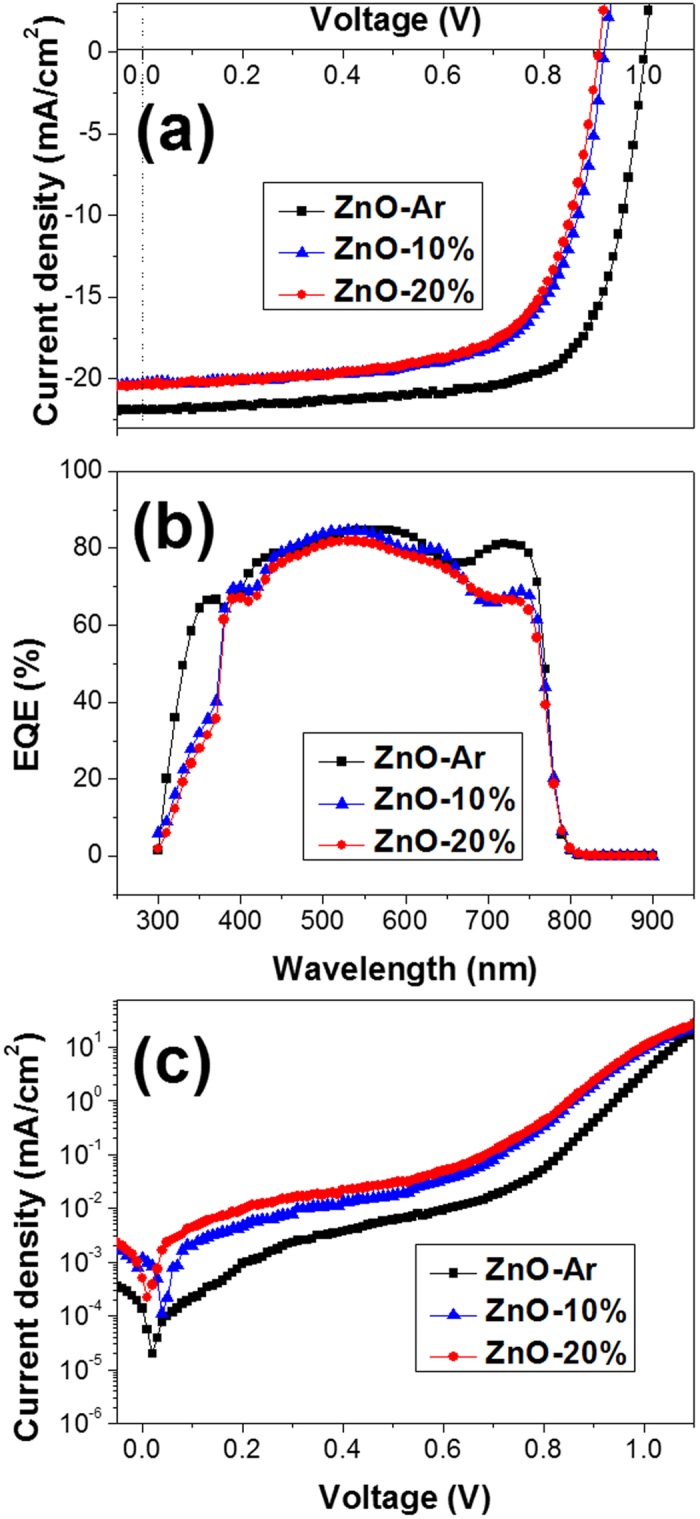
(**a**) I-V curves, (**b**) IPCE spectra, (**c**) Dark current curves of perovskite solar cell using ZnO-Ar, ZnO-10%, and ZnO-20% as the electron transport layer.

The physicochemical data discussed above confirmed that the structure, light absorption ability, morphologies and thicknesses of perovskite films (prepared *via* two-step method) deposited on all ZnO films (fabricated with and without the presence of O_2_ in working gas) are similar. However, the photovoltaic performances of the corresponding devices (the device architecture used in this study is ITO/ZnO/CH_3_NH_3_PbI_3_/spiro-OMeTAD/Ag) are significantly different. Figure 5(a) displayed the *I-V* curves of the three best devices (device with the highest efficiency) measured at a 75 ms scanning delay time using the reverse modes (from the open-circuit voltage (Voc) to the short-circuit current (Jsc)) under the illumination of 1.5 G simulated Sun light. The corresponding photovoltaic parameters are listed in Table 1. The highest PCE of the device based on CH_3_NH_3_PbI_3_ film deposited on ZnO-Ar is 15.9%. On the other hand, the device based on perovskite deposited on ZnO-10% and ZnO-20% has the PCE of only 12.8 and 12.4%, respectively. Furthermore all photovoltaic parameters of the device based on ZnO-Ar are better than those for the device based on ZnO-10% and ZnO-20% films. The statistical analysis of the photovoltaic parameters for the devices based on ZnO-Ar and ZnO-20% were also performance and listed in Table S1, ESI. Figure S4 of ESI displayed the histograms of all photovoltaic parameters for ZnO-Ar and ZnO-20% based cells. The data clearly revealed that the photovoltaic performance of perovskite solar cells based on ZnO-Ar film is better than those using ZnO-20% as an electron transporting medium.

IPCE spectra of the best cells shown in Fig. 5(b) have two troughs at around 400 nm and 600 nm, which are caused by the strong reflection of the glass/ITO substrate at these wavelength regions^30^. The integrated current density of the cells based on ZnO-Ar, ZnO-10% and ZnO-20% derived from the IPCE spectra shown in Fig. 5(b) are 20.0, 18.7 and 18.2 mAcm^−2^ respectively, which is 5 ~ 8% smaller than the Jsc obtained from the I-V curves. A difference between the current density integrated from IPCE curve and Jsc from I-V curve is generally observed in perovskite cell due to the method for measuring I-V and IPCE is not the same, see the experimental section. Device based on ZnO-Ar film has the highest IPCE values at the wavelength between 300 ~ 800 nm for the three best cells based on ZnO films fabricated at different working gas. The external quantum efficiency (EQE or IPCE value) depends on the photon absorption and carrier extraction. All Perovskite films deposited on ZnO films have similar absorption spectra implying that they have the same light harvesting capability. Therefore the difference between IPCE values is due to the difference in the carrier extraction by ZnO film. Indeed, the intensity of the photoluminescence (from the recombination of perovskite exciton) of perovskite film deposited on ZnO-Ar is significantly weaker than those for perovskite films deposited on ZnO-10% and ZnO-20% as shown in Figure S3 of ESI. The weaker PL intensity for perovskite on ZnO-Ar indicated that the electron transfer from perovskite to ZnO-Ar is more efficient. This may due to that ZnO-Ar has higher conductivity, or perovskite/ZnO-Ar has better interface contact, or the energy level of their frontier orbitals has a better match. In other words the photovoltaic performance of the devices depends on the properties of ZnO film prepared in varied working gases. This proposal was further confirmed by the dark current curves shown in Fig. 5(c). Device based on ZnO-Ar film has the smallest dark current compared to those based on ZnO-10% and ZnO-20%, suggesting that ZnO-Ar has better hole blocking ability. Since the physicochemical properties of ZnO-10% and ZnO-20% are so similar, the following studies we focus only on the differences between ZnO-Ar and ZnO-20% films.

**Figure 6:**
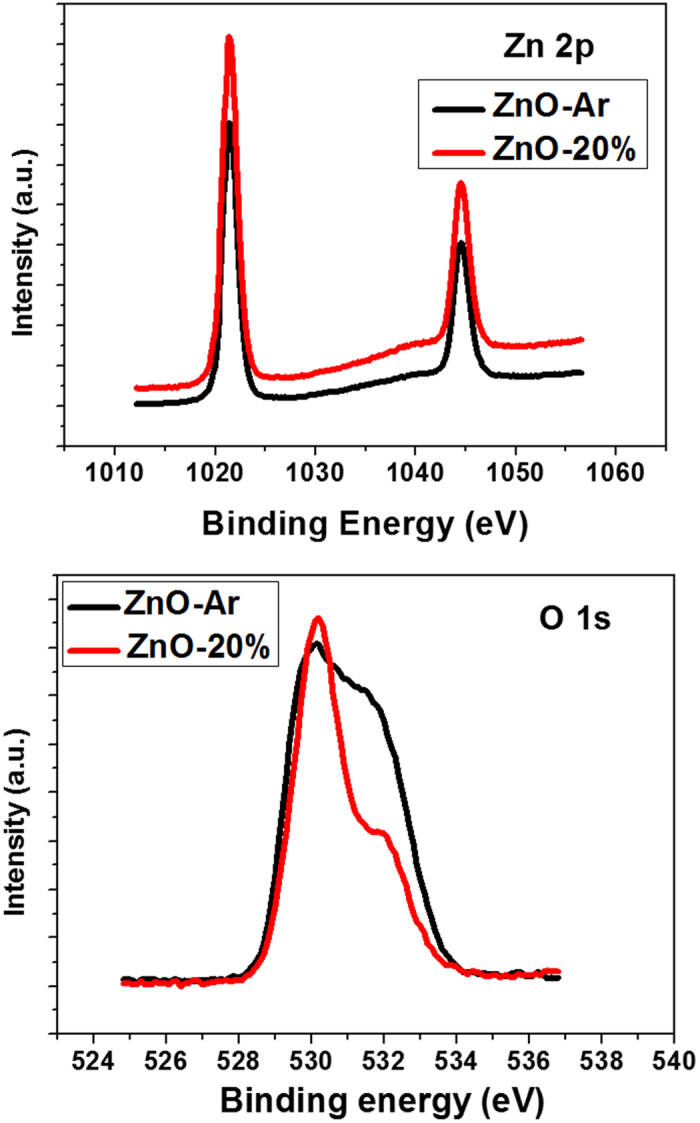
X-ray photoelectron spectroscopy (XPS) spectra of ZnO-Ar and ZnO-20% films. (top) Zn2p and (bottom) O1s.

The surface components and chemical states of ZnO films were investigated with X-ray photoelectron spectroscopy (XPS). In XPS spectra, the binding energies of all elements have been calibrated by taking the carbon C1s peak (285.0 eV) as a reference. The Zn 2p core electron for ZnO-Ar and ZnO-20% films is at the same level, having two binding energies at 1021.5 eV and 1044.5 eV (see Fig. 6(a)). Nevertheless the O_1s_ peaks (Fig. 6(b) at binding energy *ca.* 530.2 eV (belonged to O in Zn-O of the framework) for both films have a shoulder occurred at higher binding energy. This shoulder peak is associated with the oxygen-defect sites related to oxygen atom vacancies or chemisorbed oxygen in ZnO framework^31^,^32^,^33^,^34^. The O_1s_ shoulder peak of ZnO-Ar film (prepared under pure argon atmosphere) was stronger than that of ZnO-20% films (prepared under Ar/O_2_ mixed gas) suggesting that ZnO-Ar film has more defect site (most probability are oxygen vacancies since the film was prepared under no oxygen environment). More oxygen vacancies of ZnO-Ar film may result in higher conductivity. The sheet resistances of ITO, ZnO-Ar, ZnO-10% and ZnO-20% films measured with van der pauw method were 9.9, 10.6, 23.4 and 24.7 Ω, respectively. ZnO-Ar film prepared without the presence of O_2_ does have higher conductivity, due to more oxygen vacancies, consistent with the literature reports^34^,^35^,^36^,^37^. The series resistance calculated form the I-V curves of the devices base on ZnO-Ar and ZnO-10%, and ZnO-20% was also listed in Table 1. As expected, ZnO-Ar film has the highest conductivity and the corresponding cell also has the lowest series resistance. Lower series resistance of ZnO-Ar based cell is one of the reasons for having higher Voc.

**Figure 7:**
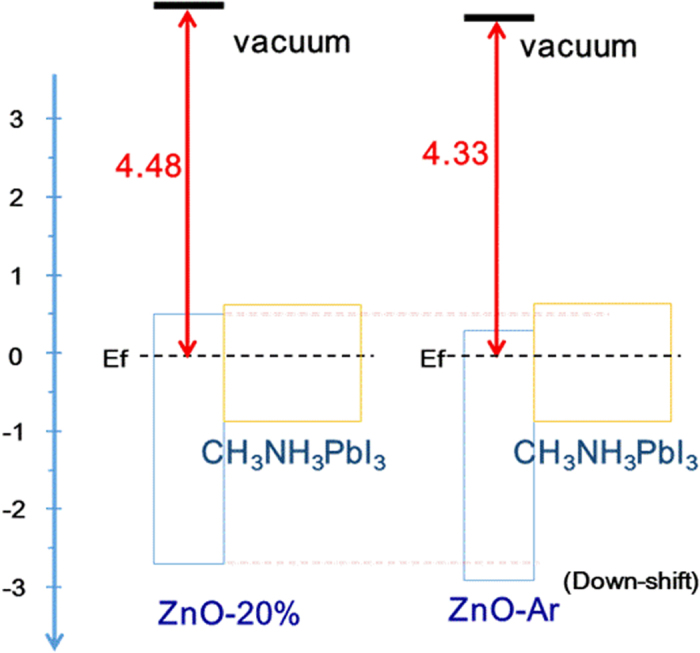
The illustration of the frontier orbitals energy levels for ZnO-Ar, ZnO-20% and perovskite.

The oxygen non-stoichiometry may down-shift the valence edge of ZnO-Ar films^34^. The band gap determined by fitting the linear regions of the square of the absorption coefficient (α^2^) versus the photon energy (displayed in Figure S12:57 PM 9/15/2015 of ESI) of ZnO-Ar and ZnO-20% films is the same, therefore the conduction band of ZnO-Ar film is also down-shift to lower energy. In order to get more information regarding to the relative energy level of the Fermi level, valence band and conduction band of ZnO films, the photoelectron spectra of ITO, ZnO-Ar and ZnO-20% were taken and displayed in Figure S5, ESI. ITO has the highest work function and the work function of ZnO-Ar is higher than that of ZnO-20%, combining the fact that ZnO-Ar and ZnO-20% have the same band gap, suggesting that ZnO-Ar has lower valence band and conduction band when perovskite film was deposited as illustrated in Fig. 7. ZnO-Ar with lower conduction band has higher driven force for the electron injection from perovskite to ZnO and lower valance edge can block the hole more efficiently. Both resulting in higher charge extraction efficient, therefore the corresponding device has higher Jsc. The hole blocking ability of ZnO-Ar film was also supported by the dark current of the corresponding device shown in Fig. 5(c). Cell based ZnO-Ar electron transporting layer has smaller dark current indicated that ZnO-Ar has better hole blocking ability when other components in both cells are supposed to be the same. Compared with ZnO-20%, ZnO-Ar has higher charge extraction efficiency, lower sheet resistance and better hole blocking ability, the resulting cell has high Jsc, Voc and FF.

**Figure 8:**
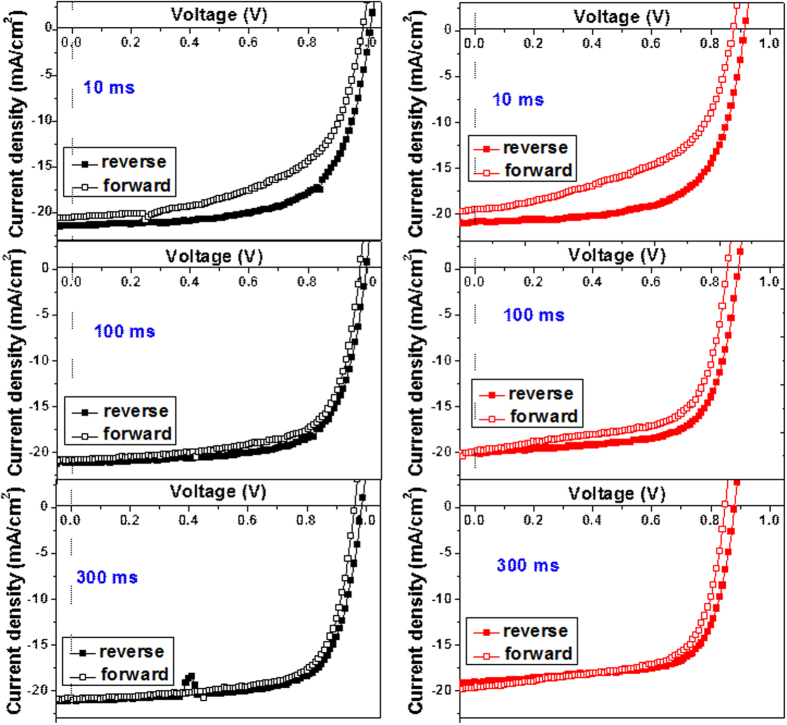
The I-V curves of the champion cell under forward and reverse scan with various delay times under AM 1.5G illumination for the devices base on ZnO-Ar (left) and ZnO-20% (right) electron transporting layers. (the delay time is defined as the time used between two data points, the data were taken every 0.01 V).

It was known that the regular perovskite solar cells exhibit a current hysteresis even at the scan rate slower than 1.0 V per second because of changing the direction of the permanent dipoles of CH_3_NH_3_^+^ under different voltage scan directions^38^. Actually even crystalline silicon solar cell has a current hysteresis when the voltage scan rate is higher than 15 V per second due to the capacitive charging and discharging effects^39^. The I-V curves of the devices based on ZnO-Ar and ZnO-20% films scan at different directions and delay times (speeds) are displayed in Fig. 8. Both devices show current hysteresis under different scan directions at scan rates from 1.0 V to 0.33 V per second and the current hysteresis decreases as the scan time decreases. Nevertheless, the device based on ZnO-Ar film shows smaller current hysteresis compared to the cell used ZnO-20% anode for all scan rates studied in this article. Snaith *et al.*^40^ has been proposed that the hysteresis is a property of the perovskite absorber or the interface between the perovskite and the charge collection layers. They measured the current hysteresis phenomenon of the conventional perovskite solar cells with several architectures and concluded that the imperfect perovskite/charge collection layer interface is the major factor causing the current hysteresis at different scan directions. We did not find any difference between the perovskite absorber deposited on ZnO-Ar and that deposited on ZnO-20%. Therefore the small degree of current hysteresis of the device based on ZnO-Ar film suggested that the charge extraction by ZnO-Ar is more efficient than that by ZnO-20%. Or the interface contact between perovskite and ZnO-Ar is better than that between perovskite and ZnO-20%. The interface contact may not mean the real physical contact but more related to the better matching of the energy level of the frontier orbitals as discussed in the previous paragraph.

## Conclusion

In conclusion, ZnO films with various oxygen deficiencies were prepared by sputtering method under different working gases. Combining with two-step spin coated perovskite film and spiro-OMeTAD hole transport layer, the resulting regular planar perovskite solar cell achieves the highest power conversion efficiency of 15.9% under AM1.5G irradiation. The electric and electronic properties of ZnO film depends on the oxygen vacancies which can be controlled by the working gases during the sputtering process. When ZnO (ZnO-Ar) film was fabricated under pure Ar, more oxygen vacancy was form in the network, which lower shifts the HOMO and LUMO energy levels, compared to that (ZnO-20%) prepared under O_2_ and Ar mixture. ZnO-Ar film is a better electron extractor and hole blocker, therefore all photovoltaic parameters of the corresponding cell are higher. These results provide a useful knowledge for preparing metal oxides to be used in the planar perovskite solar cells.

## Methods

### Materials and Physicochemical Studies

PbI_2,_ CH_3_NH_3_OH and HI(aq) were purchased from Aldrich Co. and used as received. Patterned ITO-covered glass substrates with a sheet resistance of 10-ohm were obtained from Ruilong Inc., Taiwan. CH_3_NH_3_I was synthesized with the same method published in literature^31^. Uv/Vis absorption and PL spectra were recorded with a Hitachi U-4100 and F-7000 spectrometers, respectively. The thickness of the films was measured with a depth-profile meter (Veeco Dektak 150, USA). XRD data were collected through a Brucker powder diffractometer (D8 Discover) using Cu Kα1 radiation. Scanning Electron Micrograph (SEM) was performed with a Hitachi S-4800 microscopy at 15 KV. Samples (surface and cross-section of film on the substrates) for SEM imaging were mounted on a metal stub with a piece of conducting tape then coated with a thin layer of gold film to avoid charging.

### Device Fabrication and Photovoltaic performance Measurement

A frequency (RF) magnetron sputtering method was used to prepare ZnO thin films on the patterned ITO-covered glass substrates. Sputtering was carried out in a pure argon gas and mixing argon and oxygen (O_2_/(Ar + O_2_) = 10%, 20%) atmospheres with 4-inch ZnO ceramic target. The work pressure, gas flow rate, and RF power were maintained at 4 mtorr, 100 sccm, and 80 W, respectively. For depositing perovskite layer, first, several drops of PbI_2_ precursor solution (1.0 M in DMF) were spin-coated on the top of ZnO coated ITO at a spin rate of 3000 rpm. After PbI_2_ layer being dried, CH_3_NH_3_I solution (50 mg in 1.0 mL isopropanol) was spin-coated on top of PbI_2_ film at spin rates of 3000 rpm, 15 sec to form perovskite structure. More detailed information about the two-step spin-coating method can be found in literature^24^. After perovskite film was formed, 2,2′,7,7′-tetrakis[N,N-di(4-methoxyphenyl)amino]-9,9′-spirobifluorene (spiro-OMeTAD) used as the hole transport layer was then deposited by spin-coating at 4,000 rpm for 30 s. The spiro-OMeTAD precursor solution was prepared by dissolving 80 mg spiro-OMeTAD, 28.5 μL 4-tert-butylpyridine and 17.5 μL lithium bis(trifluoromethyl- sulphonyl)imide solution (520 mg in 1.0 mL acetonitrile) in 1 mL chlorobenzene. The fabrication procedures were carried out in the ambient atmosphere at low temperature (<100 °C). Finally, the device was transferred to a vacuum chamber for coating Ag electrode (100 nm). The effective area of the device was 0.1 cm × 0.2 cm defined by a non-reflective metal mask. J-V characteristics of the cells were taken using a Keithley 4200 source measuring unit under a simulated AM1.5G light (Wacom solar simulator) at 100 mAcm^−2^. The intensity of the simulated AM1.5G light was calibrated by KG-5 Si diode. External quantum efficiency (EQE) or incident photo-to-current conversion efficiency (IPCE) was measured in air. A chopper and lock-in amplifier were used for the phase sensitive detection with QE-R3011 measurement system (Enlitech Inc., Taiwan).

## Additional Information

**How to cite this article**: Tseng, Z.-L. *et al.* Surface Engineering of ZnO Thin Film for High Efficiency Planar Perovskite Solar Cells. *Sci. Rep.* **5**, 13211; doi: 10.1038/srep13211 (2015).

## Supplementary Material

Supplementary Information

## Figures and Tables

**Table 1:** Photovoltaic characteristics of the planner perovskite solar cell based ZnO anode prepared under different atmosphere.

Working gas	V_OC_ (V)	J_SC_(mAcm^−2^)	FF (%)	PCE (%)	Rs (Ohm-cm^2^)
pure argon	1.00	21.8	72.6	15.9	3.2
O_2_/(Ar + O_2_) = 10%	0.92	20.3	68.1	12.4	3.7
O_2_/(Ar + O_2_) = 20%	0.90	20.3	67.6	12.4	3.7
