# Supplementary material for: Surface Engineering of ZnO Thin Film for High Efficiency Planar Perovskite Solar Cells
Source: Sci Rep. 2015 Sep 28;5:13211. doi: 10.1038/srep13211 (PMC4585934; doi:10.1038/srep13211)
Supplement: Supplementary Information [file srep13211-s1.pdf]

Supporting Information

Surface Engineering of ZnO Thin Film for High Efficiency Planar Perovskite Solar Cells

Zong-Liang Tseng[b], *Chien-Hung Chiang*[b], *and Chun-Guey Wu*[a,b*]

[a]*Department of Chemistry and* [b]*Research Center for New Generation Photovoltaics, National Central University, Jhong-Li, 32001, Taiwan, ROC.*

E-mail address of Professor C. G. Wu: t610002@cc.ncu.edu.tw

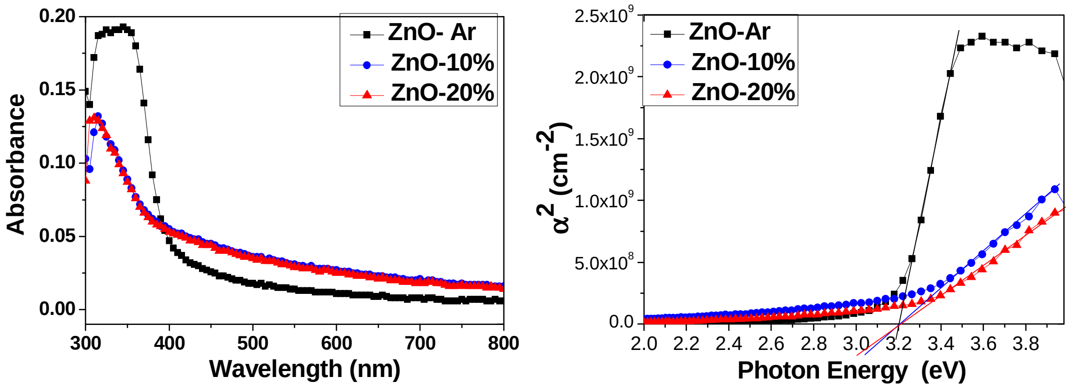

Figure S1: UV/Vis absorption (left) and band gap (b) of ZnO films.
(The optical band gap (Eg) was determined by fitting the linear regions of the square of the absorption coefficient (α^2) versus the photon energy (hν).)

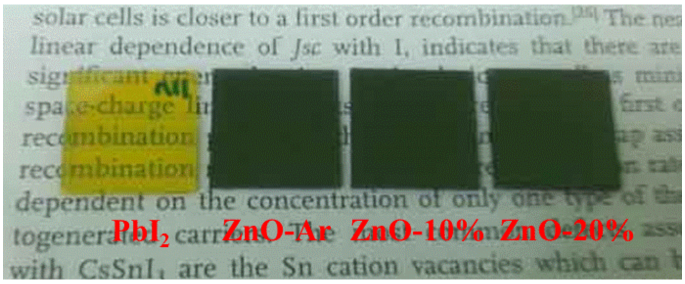

Figure S2: The picture (taken by Z.-L. Tseng) of PbI_2 film on ZnO-Ar (left) and perovskite film deposited on top of ZnO-Ar ZnO-10%, and ZnO-20% (three in the right side).

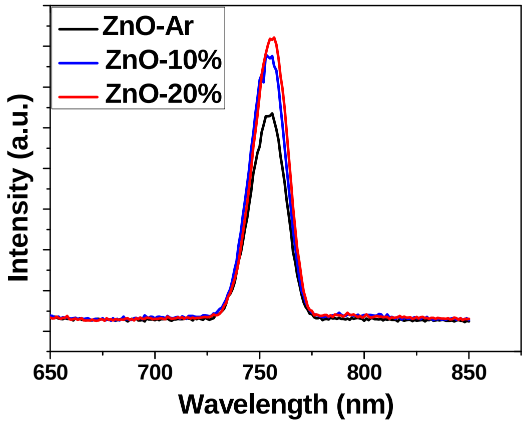

Figure S3: The PL spectra of perovskite/ZnO-Ar, perovskite/ZnO-10%, and perovskite/ZnO-20% films

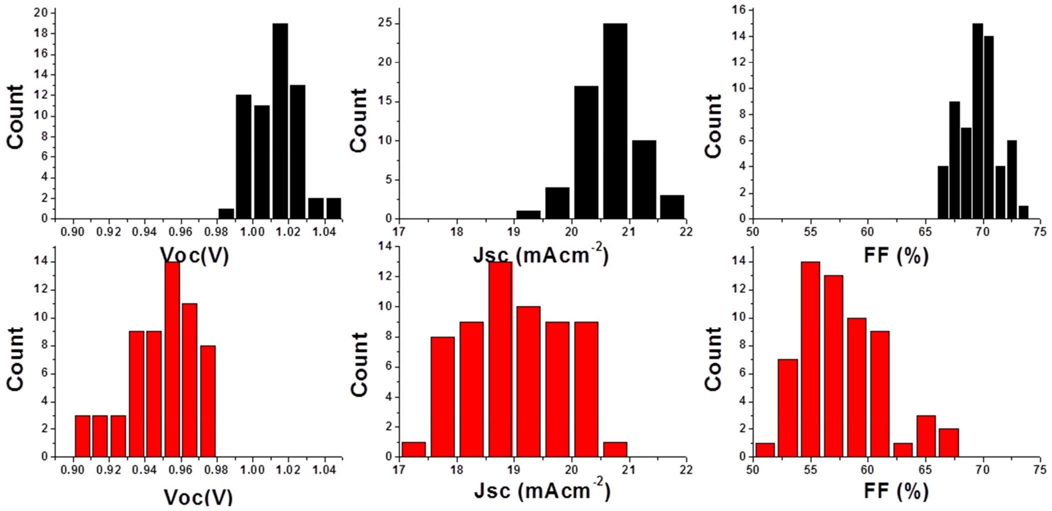

Figure S4: The histograms of Jsc, Voc and FF of 60 devices based on ZnO-Ar (top) and ZnO-20% (bottom) films.

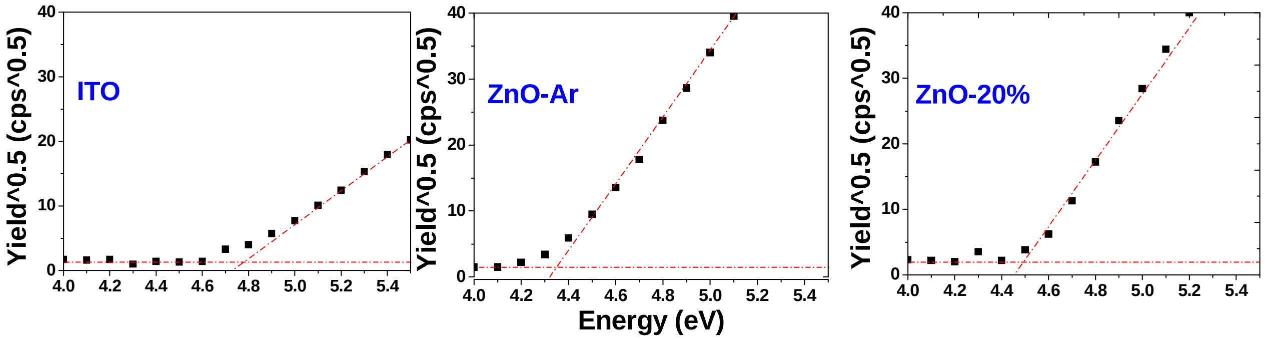

Figure S5: Photoelectron spectra of ITO, ZnO-Ar and ZnO-20% films

Table S1. The average of the photovoltaic characteristics of the planner perovskite solar cell based ZnO anode prepared under different atmosphere.

**Working gas**	V_{OC} (V)	**J_{SC} (mAcm^{-2})**	FF (%)	PCE (%)	Rs (Ohm-cm^2)
**pure argon**	**1.01±0.012**	**20.6±0.497**	**69.5±-1.72**	**14.5±-0.55**	**4.09±0.473**
**O_2/(Ar+O_2) = 20%**	**0.948±0.021**	**19.0±0.873**	**57.6±3.62**	**10.4±0.85**	**7.73±1.26**

(± represents the stand deviation)

The number of devices is 60.

herefore the small degree of current hysteresis of the device based on ZnO-Ar film suggested that the charge extraction by ZnO-Ar is more efficient than that by ZnO-20%.
